# Expression of retinoic acid-binding proteins and retinoic acid receptors in sebaceous cell carcinoma of the eyelids

**DOI:** 10.1186/s12886-015-0145-5

**Published:** 2015-10-26

**Authors:** Yueh-Ju Tsai, Shu-Ya Wu, Hsuan-Ying Huang, David Hui-Kang Ma, Nan-Kai Wang, Ching-Hsi Hsiao, Ching-Yi Cheng, Lung-Kun Yeh

**Affiliations:** Department of Ophthalmology, Linko Chang-Gung Memorial Hospital, Taoyuan, Taiwan; College of Medicine, Chang-Gung University, Taoyuan, Taiwan; Department of Pathology, Kaohsiung Chang-Gung Memorial Hospital, Kaohsiung, Taiwan; Graduate Institute of Health Industry Technology, Research Center for Industry of Human Ecology, Chang Gung University of Science and Technology, Taoyuan, Taiwan

**Keywords:** Retinoic acid (RA) signaling, Sebaceous cell carcinoma

## Abstract

**Background:**

Sebaceous cell carcinoma of the eyelid is a malignant tumor. However, the pathoetiology of sebaceous cell carcinoma is not clear. Retinoic acid (RA) signaling is essential for skin epidermal differentiation including the eyelids. In this study, we investigate the expression of β-catenin, RA-binding proteins and RA receptors in sebaceous cell carcinoma of the eyelid and try to estimate their influence on its pathoetiology.

**Methods:**

Retrospective, noncomparative, consecutive interventional case series. Sixteen cases of eyelid sebaceous gland carcinoma who received tumor excision at our hospital between 2001 and 2011 were included. Immunohistochemical staining for β-catenin, cellular retinoic acid binding protein 1 (CRABP1), cellular retinoic acid binding protein 2 (CRABP2), fatty acid-binding protein 5 (FABP5), retinoic acid receptors (RAR-α, −β, −γ), and retinoid X receptors (RXR-α, −β, −γ) was performed on tissue samples obtained from tumor excision.

**Results:**

Of the 16 sebaceous cell carcinoma cases reviewed, six were male and 10 female. The mean follow-up period was 6.7 ± 3.66 years (range, 0.3–13 years). Of these 16 cases, the expression of β-catenin was significantly increased in sebaceous cell carcinoma cases. CRABP1 was similarly expressed in the sebaceous cell carcinoma and control groups. CRABP2 and FABP5 were expressed in hair follicles of lid skin in both groups, whereas the CRABP2 and FABP5 were aberrantly expressed in the tumor cells of the sebaceous glands. Notably, the expression of retinoic acid receptor (RAR-β) and retinoid X receptors (RXR-β, −γ) was significantly upregulated in sebaceous cell carcinoma of the eyelids.

**Conclusions:**

Our findings indicate that retinoic acid signaling is related to the pathogenesis of sebaceous cell carcinoma of the eyelids.

## Background

Sebaceous cell carcinoma (SeCC) of the eyelid is a highly aggressive malignant tumor that arises from the meibomian glands, Zeiss glands of the eyelid, or sebaceous glands of the caruncle [1]. Sebaceous cell carcinoma is more frequent in Asians than in Caucasians. In the West it accounts for only 0.2–4.7 % of all eyelid malignancies [2] whereas in Asia it accounts for 11–33 %. The prevalence of SeCC is 8–23 % in Taiwan. Sebaceous cell carcinoma is notorious for masquerading as other benign and malignant lesions, often resulting in delayed diagnosis. In addition, diffuse epithelial involvement (pagetoid growth pattern) is present in about half of the cases. Therefore, it is usually associated with a high risk of recurrence and metastatic diseases. Other factors associated with a poor prognosis include vascular, lymphatic or orbital invasion, involvement of both upper and lower eyelids, poor differentiation, multi-centric origin, highly infiltrative pattern, and tumor size more than 2 cm. Etiological risk factors of SeCC include radiation exposure, advanced age, and a genetic predisposition to Muir–Torre syndrome, which includes sebaceous adenoma and sebaceous carcinoma [2–5].

Unfortunately, the prognosis of SeCC is unpredictable. To date, there are no specific reliable markers available for predicting the prognosis of the patients. The retinoids have been reported to affect the growth and differentiation of epithelial tissue and to play an important role in essential biologic processes, differentiation, proliferation, and apoptosis [6–10]. Furthermore, retinoids, whose biological functions are mainly mediated by retinoid receptors, have been used as a therapy for some non-melanoma skin cancers [11]. The cellular retinoic acid-binding proteins (CRABPs) may regulate the accessibility of retinoic acid (RA) to the RA receptors and are thought to affect the prognosis of cancer. In particular, CRABP1 is expressed in hair follicles of normal skin (dermal papilla) and in the stroma of epidermal tumors. A member of the fatty acid-binding protein (FABP) family, FABP5, is found abundantly in skin epidermal cells, adipocytes, macrophages, liver, heart, sebaceous glands, and anagen follicle bulbs [11, 12]. Previous experiments indicate that CRABP2 and FABP5 are abundantly expressed in the differentiating cells of sebaceous glands, interfollicular epidermis, and hair follicles [11]. Therefore, combinations of RA receptors and CRABPs may be an important factor in mediating the effects of RA on transcription and cellular processes. Moreover, the dynamic patterns of expression of CRABPs also reflect cross talk of RA and Wnt/β-catenin signaling in different developmental and homeostatic situations [11, 13–17]. Aberrant expression of β-catenin has been reported in different tumors such as colorectal, hepatocellular, breast carcinoma, oral squamous cell carcinoma, and non-melanoma skin tumors [18]. The RA receptors are nuclear receptors related to the steroid and thyroid hormone receptors. So far, two classes of nuclear retinoid receptors (RARs and RXRs) have been reported, and each has three main subtypes, −α, −β, and -γ. They play a pivotal role as ligand-dependent transcription factors. RA receptors act in heterodimeric combinations with retinoid X receptors (RXRs) and facilitate DNA binding of the RAR–RXR complex [19]. RA signaling is mediated by RA binding to RARs, which form heterodimers with RXRs, and is regulated by RA-binding proteins [19, 20]. Retinoids, that are Vitamin A derivatives, as ligands for binding to these nuclear receptor transcription factors (RARs and RXRs), are strongly associated with the development of skin cancer and its subsequent prognosis.

Therefore, we aimed to investigate the role of RA signaling pathway in the pathogenesis of SeCC. In this study, we retrospectively analyzed the variation in the immunohistochemical expression of related signaling pathway binding proteins between SeCC cases and normal control cases, and tried to explore the role of RA pathway in the pathogenesis of SeCC.

## Methods

### Ethics statement, subject recruitment, and clinical assessment

No animal experiments were conducted in this study. This retrospective study to examine human eyelid tissues with sebaceous cell carcinoma was approved by the Institutional Review Board of Chang Gung Memorial Hospital, Linko, Taiwan (registry numbers 100-3459B). All procedures conformed to the Declaration of Helsinki and the ARVO statement for research involving human subjects. Written consent was obtained before use of the patients’ tissues for histological study. Nineteen eyelids, 16 from patients with sebaceous cell carcinoma of eyelids and three from patients with conjunctival nevus, were included in this study. Sebaceous cell carcinoma was classified according to the 7^th^ edition eyelid carcinoma classification system from the American Joint Committee on Cancer. The clinical history and follow-up duration for each evaluated case was recorded.

### Patient demographics and clinical features

**Fig. 1:**
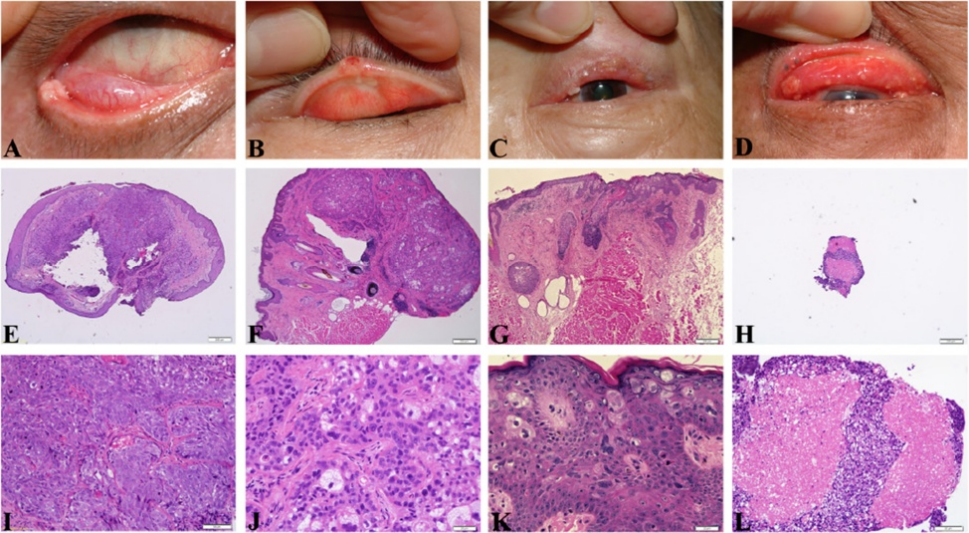
External eye photos and hematoxylin-eosin staining of sebaceous cell carcinoma (SeCC) of eyelids. **a**-**d**: Preoperative photos show (**a**) A fleshy mass arising from the lower eyelid tarsus (Case 2), (**b**) A small ulcerated nodule at the right upper eyelid (Case 6), (**c**) An ill-defined lesion involving the upper eyelid, masquerading as blepharitis (Case 12), (**d**) An advanced tumor diffusely infiltrative growth that involves the whole upper tarsus (Case 16). **e**-**h**: The corresponding scanning views (4×) of hematoxylin-eosin sections of the eyelid-conjunctiva specimens labled as A-D in the upper row. **i**-**l**: Higher power magnification of scanning views in the middle row shows (**i**) solid sheets of pleomorphic carcinoma cells without apparent sebaceous differentiation (×20), (**j**) irregular and interconnecting nests of carcinoma cells with foamy cytoplasmic droplets indicative of sebaceous differentiation (×40), (**k**) intraepithelial (pagetoid) growth of sebaceous carcinoma cells involving the epidermis (×20), (**l**) a comedo pattern of sebaceous carcinoma showing central necrosis (×20)

From 2001 to 2011, six male patients and 10 female patients, with a female preponderance (M: F ratio of 0.6), were included in the SeCC group in this study. The mean age was 66.4 ± 2.28 years (range, 46–77 years), and the median follow-up duration was 6.7 ± 3.66 years (range, 0.3–13 years). One male patient and two female patients were included in the control group. The mean age was 78.7 ± 0.33 years (range, 78–79 years). The general information and clinical data of patients with sebaceous cell carcinoma were listed in Table 1. Clinical images of sebaceous cell carcinoma of eyelids (SeCC) were presented at Fig. 1.

**Table 1:** General information and clinical data of patients with sebaceous cell carcinoma

No.	Primary surgery	Primary staging		Subaequent follow-up					F/U	Mortality
		Staging	TNM	Local recur	LN meta	Distant meta	Staging	Mx		
1	Hughs	IC	T2bN0M0	N	Y	N	III B	Surgery + R/T	8.5 Ys	N
2	Tenzel	IB	T2aN0M0	U	U	U			0.3 Ys	U
3	Tenzel	IC	T2bN0M0	N	N	N		Observation	1.3 Ys	N
4	Tenzel	IB	T2aN0M0	Y	N	N	II	Local excision	8.5 Ys	Y
5	Tenzel	IB	T2aN0M0	Y	N	N	IC	Local excision	6 Ys	Y
6	Tenzel	IB	T2aN0M0	N	N	N		Observation	8 Ys	N
7	Tenzel	IB	T2aN0M0	Y	Y	Y	IV	Exenteration	8 Ys	Y
8	Tenzel	IB	T2aN0M0	N	N	N		Observation	13 Ys	N
9	Tenzel	IB	T2aN0M0	Y	N	N	II	Local excision	11 Ys	N
10	Hughs	II	T3aN0M0	Y	Y	Y	IV	C/T	1.5 Ys	Y
11	Tenzel	IB	T2aN0M0	Y	N	N	III A	Exenteration	9.5 Ys	N
12	Cutler-beard	IC	T2bN0M0	Y	Y	N	III B	C/T	8 Ys	Y
13	Cutler-beard	IC	T2bN0M0	N	N	N		Observation	8 Ys	N
14	Cutler-beard	IC	T2bN0M0	Y	N	N	II	Local excision	4 Ys	N
15	Tenzel	IB	T2aN0M0	N	N	N		Observation	8.5 Ys	N
16	Tarsus replaced	III A	T3bN0M0	Y	Y	Y	IV	R/T+ C/T	3 Ys	Y

No.: Patient number; C/T: chemoterapy; R/T: radiation therapy

Conventional surgery remained the main treatment modality for sebaceous cell carcinoma. To minimize the risk of recurrence, complete excision with allowing a wide tumor-free margin 3–5 mm was essential for SeCC cases. At the time of primary excision, or for subsequent excision of residual tumor, the margins were checked with frozen section technique. Planned surgical reconstruction of the defect was then undertaken. Complete resection and primary closure could be accomplished for the defects less than 10 mm. Tenzel rotational flap was often necessary to mobilize enough skin to facilitate wound closure for the defects around half of the length of eyelid. Larger defects might need lid-sharing procedure from the opposite lid. Conjunctival and tarsal replacement using tarsal flaps or grafting with oral mucosa or hard palate graft, combined with topical chemotherapy stood as a conservative treatment in cases that were not far advanced. Exenteration was justified for advanced diffuse disease with anterior orbital soft tissue invasion.

### Immunohistochemistry

Immunohistochemical analysis was performed to determine the expression of the proteins of interest in tissue sections. All samples had been fixed with 4 % paraformaldehyde in phosphate buffered saline soon after tumor excision and embedded in paraffin. Then samples were cut into 4 μm thick sections and mounted on glass slides. After deparaffinization, rehydration, and blocking with 5 % goat serum for 1 h, the sample slides were incubated at 4 °C overnight with the following primary antibodies (Table 2), including antibodies against β-catenin, cellular retinoic acid-binding proteins (CRABP1, CRABP2), fatty acid-binding protein 5 (FABP5), retinoic acid receptors -α, −β, and -γ (RAR-α, −β, −γ), and retinoid X receptors -α, −β, and -γ (RXR-α, −β, −γ). The slides incubated with irrelevant immunoglobulin (IgG) were used as negative controls. All the primary antibodies used in this study were known to be specific to target proteins, and no obvious cross-reactions with any other proteins were noted. After washing, the sections were incubated at room temperature for 1 h with appropriate secondary antibodies, including goat anti-rabbit IgG-B (β-catenin, CRABP2, FABP5, RAR-β, RAR-γ), goat anti-mouse IgG-B (CRABP1), and donkey anti-goat IgG-B (RAR-α). Non-specific reactions were blocked by incubating sample slides with 2.5 % bovine serum albumin (BSA) in PBS for 30 min. At the end, the samples were observed by microscope and photographed. Tissue sections were also processed for routine hematoxylin- eosin (H & E) staining for morphological comparison.

**Table 2:** Primary antibodies, concentration and sources

Antibody	Category	Dilution	Source
*β-*catenin	Rabbit polyclonal	1: 2000	Sigma, St. Louis, USA
CRABP-1	Mouse monoclonal	1: 1000	Abcam, Cambridge, England
CRABP-2	Rabbit polyclonal	1: 100	Abcam, Cambridge, England
FABP-5	Rabbit polyclonal	1: 400	Abcam, Cambridge, England
RAR-*α*	Goat polyclonal	1: 160	Abcam, Cambridge, England
RAR-*β*	Rabbit polyclonal	1: 50	Abcam, Cambridge, England
RAR-γ	Rabbit polyclonal	1: 100	Bioss, Woburn, USA
RXR-*α*	Rabbit polyclonal	1: 250	Abcam, Cambridge, England
RXR-*β*	Mouse monoclonal	1: 100	Abcam, Cambridge, England
RXR-γ	Rabbit polyclonal	1: 100	Abcam, Cambridge, England

## Results

### Surgical outcome

At initial presentation, there were nine patients staging as T2a, five patients staging as T2b, one patient staging as T3a, and one patient staging as T3b. None of our SeCC patients received primary exenteration; five patients underwent wide surgical excision with the opposite lid sharing procedure, 10 patients underwent wide excision and local reconstruction with Tenzel rotational flap, and one received total replacement of tarsus due to diffuse tarsal involvement (Case 16). Local tumor recurrence was noted in nine patients during the follow-up period. No lymph node metastasis was detected in our SeCC patient group at the time of diagnosis, and subsequent lymph node metastasis was found in five cases; distant metastasis was found in three patients during the follow-up period. There were no significant differences regarding patient’s demographic features, including age and sex, or follow-up periods between the group with metastasis and the group without metastasis (Table 1). Fig. 1 presents the clinical and pathological images of the SeCC cases. Pre-operative clinical photograph showed a fleshy mass arising from the lower eyelid margin of a 68-year-old male (Case 2, Fig. 1a). Fig. 1b presented a nodule of sebaceous carcinoma at the right upper eyelid of a 54-year-old female (Case 6). No local recurrence had been noted after 8-year follow-up. Figure 1c presented an ill-demarcated lesion diffusely involving the left upper eyelid of a 74-year-old female, masquerading as blepharitis (Case 12). Fig. 1d showed an advanced sebaceous carcinoma case involving the whole right upper tarsus of a 71-year-old male, who died 3 years after due to systemic metastasis (Case 16). Tissue sections for routine H & E staining were shown in Fig. 1e-l.

### Immunohistochemistry

**Fig. 2:**
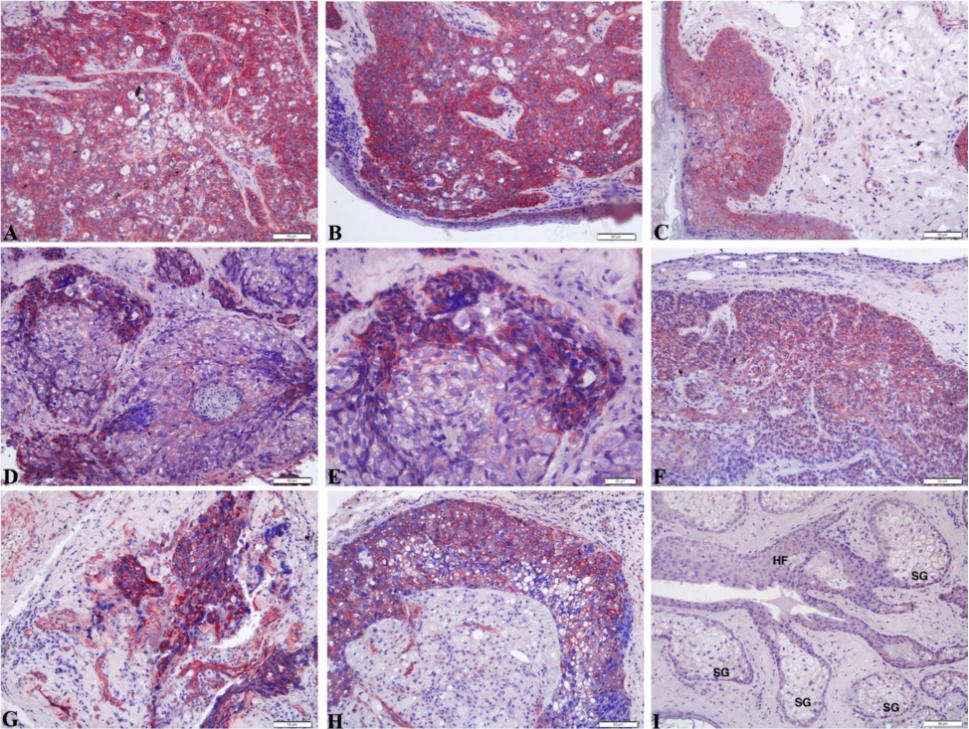
Immunohistochemistry shows the expression of β-catenin in sebaceous cell carcinoma. Aberrant expression of β-catenin in SeCC cases. No nuclear staining of β-catenin was observed in any of these SeCC cases. (**a**,**b**) case 6; (**c**) case 12; (**d**,**e**) case 8; (**f**,**g**) case 16; (**h**) case 15; (**i**) control case: HF (hair follicles); SG (sebaceous gland cells). Scale bars represent 50 μm

β-Catenin, a transcription factor of the *Wnt* signaling pathway, is a component of the adherens junction. It supports Ca^2+^-dependent cell-to-cell contact for adhesion, and plays a role in both signal transmission and anchoring the actin cytoskeleton. Most specimens presented a membranous accumulation of β-catenin with some elevated cytoplasmic levels in 81 % of the SeCC cases (*n* = 13/16, 81 %) (Fig. 2). The aberrant membranous-cytoplasmic expression of β-catenin was found in SeCC cases (Fig. 2a–h) compared with control case (Fig. 2i). No nuclear staining of β-catenin was observed in any of the SeCC cases. Therefore, the membranous-cytoplasmic overexpression of β-catenin in some cases of SeCC may be caused in part by dysregulation of the *Wnt*/β-catenin pathway.

**Fig. 3:**
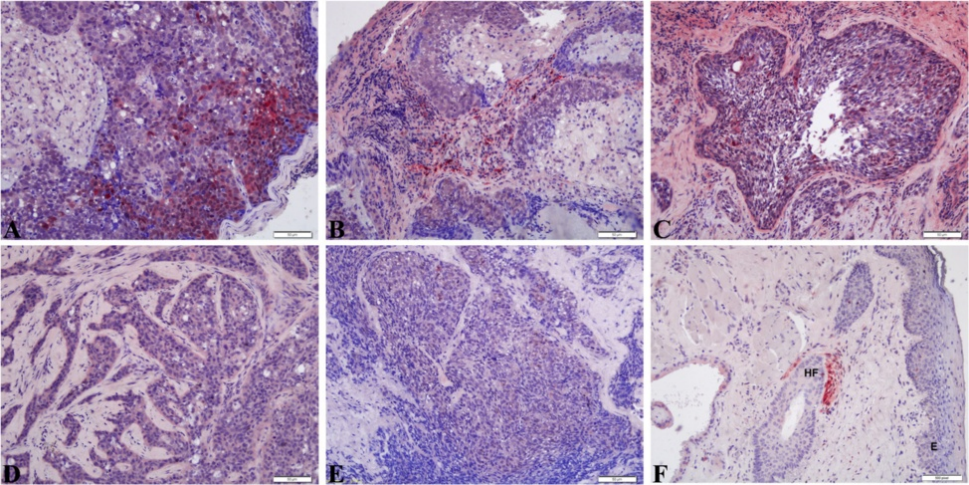
Immunohistochemistry shows varying expression of CRABP1 in sebaceous cell carcinoma. (**a**,**b**) case 16; (**c**) case 10; (**d**) case 6; (**e**) case14; (**f**) control: E (epithelium), HF (hair follicle). Scale bars represent 50 μm

**Fig. 4:**
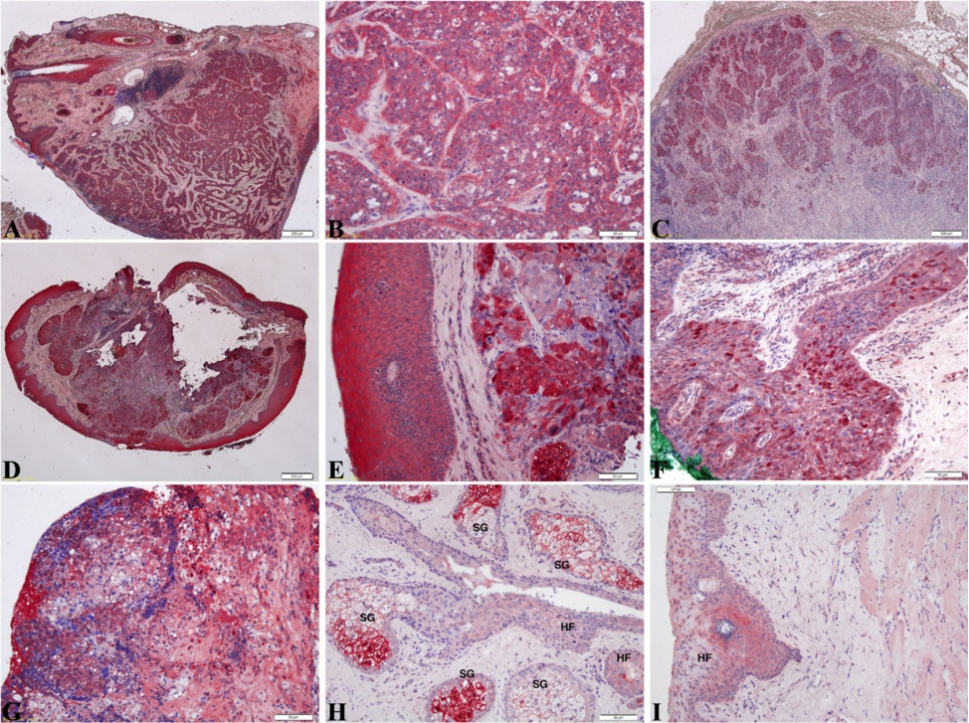
Immunohistochemistry shows the overexpression of CRABP2 in sebaceous cell carcinoma. (**a**,**b**) case 6; (**c**) case 7; (**d**,**e**) case 2; (**f**) case 11; (**g**) case 16; (**h**, **i**) control: CRAMP2 is expressed in the differentiated sebaceous gland cells (SG), and hair follicles (HF). Scale bars represent 50 μm

Figures 3 and 4 showed the CRABP1 and CRABP2 proteins were expressed in tumor cells of the SeCC cases, respectively. Figure 3a-e presented that the CRABP1 protein was expressed in tumor cells of the SeCC cases (*n* = 12/16, 75 %) with mild to moderate intensity (Fig. 3a-e). Fig. 4a-g showed that the aberrant expression of the CRABP2 protein was found in the tumor cells of SeCC cases (*n* = 15/16, 93.75 %) with moderate to strong intensity, whereas the CRABP2 protein was only mild-to-moderate weak expression in the control cases (Fig. 4h, i).

**Fig. 5:**
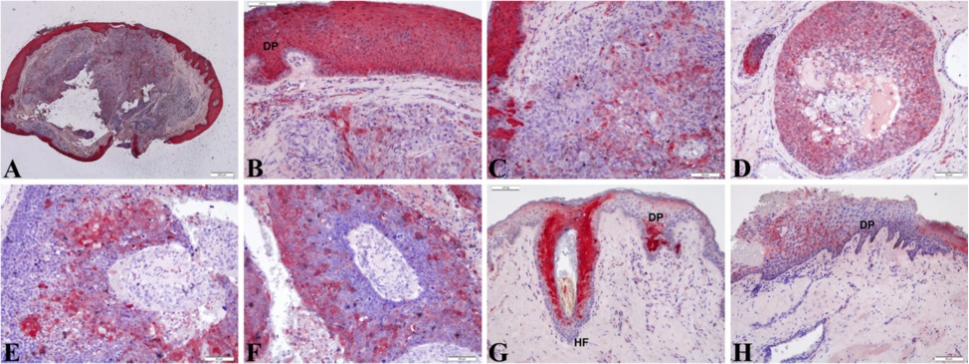
Immunohistochemistry shows the expression of FABP5 in sebaceous cell carcinoma. Aberrant expression of FABP5 in SeCC cases was shown not only in dermal papillae (DP) but also in the SeCC tumor cells, whereas in control cases the expression was seen only in dermal papillae (DP) and the hair follicles (HF). (**a**,**b**,**c**) case 2; (**d**) case 12; (**e**,**f**) case 9; (**g**,**h**) control. Scale bars represent 50 μm

Figure 5 showed the expression of FABP5 protein was aberrantly expressed with moderate to strong intensity in the SeCC cases not only in hair follicles but also in the SeCC tumor cells (*n* = 10/16, 65 %), whereas the FABP5 protein in control cases was only focus in hair follicles.

**Fig. 6:**
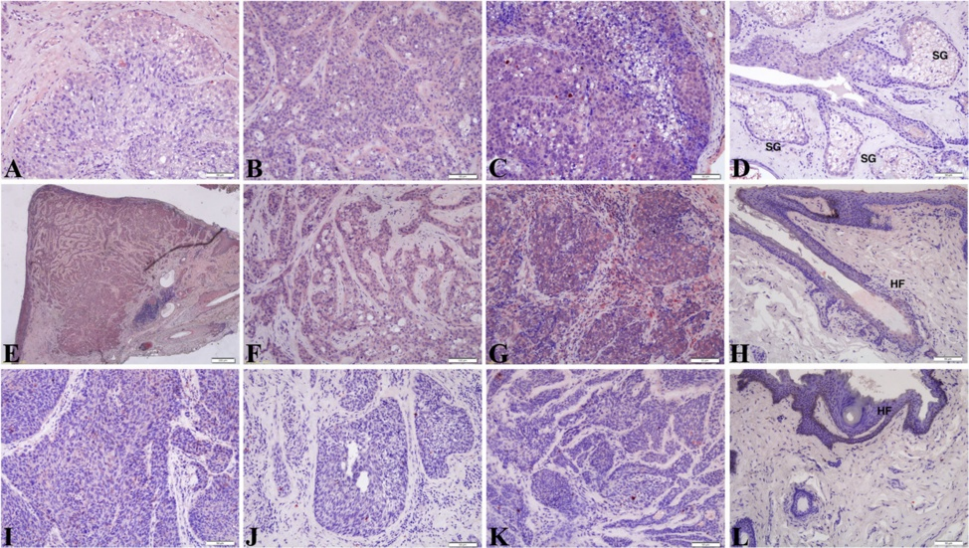
Immunohistochemistry shows the expression of retinoic acid receptors (RAR-α, −β, −γ) in SeCC cases. **a**–**d**: The expression of RAR-α was weakly positive in both groups. (**a**) case 2; (**b**) case 6; (**c**) case16; (**d**) control. **e**–**h**: Aberrant expression of RAR-β was observed in the SeCC cases on the tumor cells. (**e**, **f**) case 6; (**g**) case 7; (**h**) control; **i**-**l**: The expression of RAR-γ was weak-to-no staining in both groups. (**i**) case 3; (**j**) case 10; (**k**) case 15; (**l**) control . Scale bars represent 50 μm

**Fig. 7:**
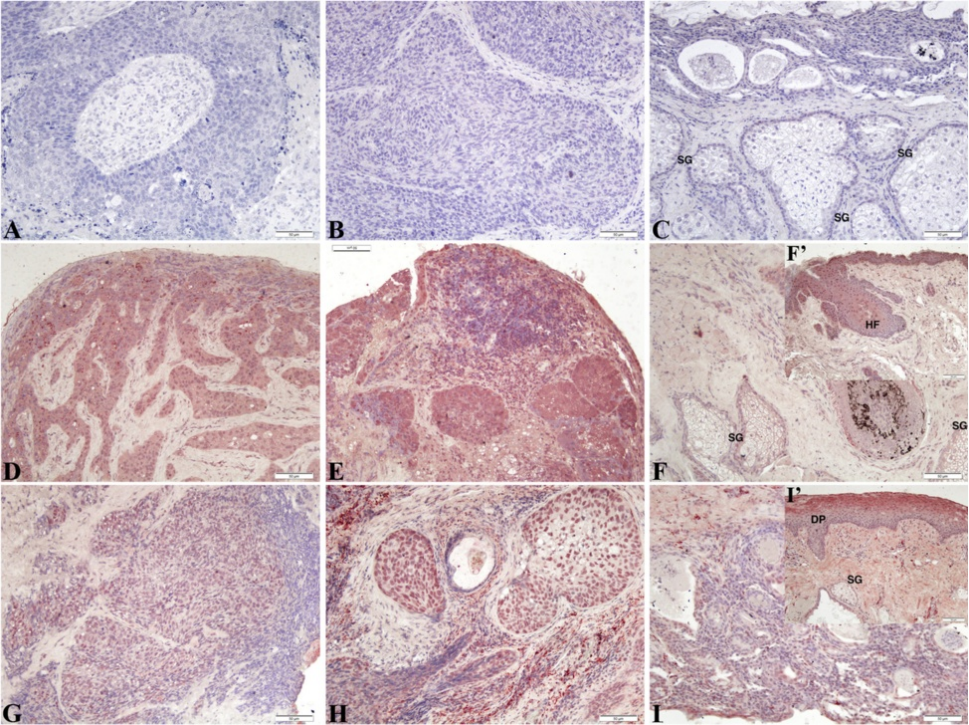
Immunohistochemistry shows the expression of retinoic X receptors (RXR-α, −β, −γ) in SeCC cases. **a**–**c**: The expression of RXR-α was negative in both groups. (**a**) case 9; (**b**) case 3; (**c**) control. **d**–**f**: The expression of RXR-β was observed in the SeCC cases. (**d**) Case 6; (**e**) Case 16; (**f**) control; (**f**’) control. **g**–**i**: The expression of RXR-γ was observed in the SeCC cases. (**g**) Case 14; (**h**) Case 11; (**i**) control; (**i**’) control. Scale bars represent 50 μm

The expression of retinoic acid receptors (RAR-α, −β, −γ) and retinoic X receptors (RXR-α, −β, −γ) were shown in Figs. 6 & 7, respectively. Figure 6a-c showed the expression of RAR-α receptor was mildly increased in SeCC cases (*n* = 15/16, 93.75 %). However, the expression of RAR-α was weakly positive in both SeCC and control cases without significant differences. Aberrant expression of RAR-β was observed in the SeCC cases on the tumor cells (*n* = 13/16, 81.25 %; Fig. 6e-g), whereas the expression of RAR-γ was weak-to-no in SeCC cases (*n* = 14/16, 87.5 %; Fig. 6i-k). Therefore, the expression of RAR-α and RAR-γ was similarly weakly positive with no significant difference in either group (Fig. 6a-c; i-k). The expression of RAR-β was significantly upregulated in the SeCC group (Fig. 6e-g). Fig. 7 showed the expression of RXR-α receptor showed a negative pattern in 87.5 % of SeCC cases (*n* = 14/16), which was similar to normal tissue without significant differences (Fig. 7a-c). The expression of RXR-β was increased obviously, with mild to moderate intensity in SeCC cases as shown in Fig. 7d, e (*n* = 13/16, 81.3 %). The RXR-γ expression presented with mild to moderate intensity in SeCC cases (*n* = 6/16, 93.75 %; Fig. 7g, h). In summary, the expression of RAR-β and RXR-β, −γ receptors were significantly upregulated during the development of sebaceous cell carcinoma cases.

## Discussion

In this study, we present the immunohistochemical expression of β-catenin, RA signaling molecules (CRABP1, CRABP2, FABP5), and related retinoic acid receptors (RARs and RXRs) in SeCC cases. Multiple predisposing factors have been proposed, such as radiation exposure, advanced age, race, and genetic predisposition [3–5]. However, the exact pathoetiology of SeCC remains unknown. As there are no available specific markers for predicting prognosis, the course of SeCC is difficult to predict. Collins and Watt proposed that dynamic patterns of CRABP expression reflect cross talk of RA and W*nt*/β-catenin signaling in different developmental and homeostatic situations of skin [11]. Zhang, Liu and co-workers proposed that conditional expression of a murine C*tnnb1* gene gain-of-function mutation alone caused corneal neoplasia and neovascularization, resembling human ocular surface squamous neoplasia (OSSN) [20]. In particular, human OSSN patients exhibited nuclear translocation of β-catenin. These results indicated that β-catenin activation might have an important role in tumorigenesis, resulting in oncogenic transformation. Sen and colleagues showed that cytoplasmic overexpression of β-catenin found in the majority of cases of SeCC (66 %) of eyelid which was significantly related to tumor size [21]. Therefore, they proposed that β-catenin overexpression in SeCC may be due to dysregulation of the W*nt*/β-catenin pathway [21]. However, its role in the pathoetiology and prognosis of sebaceous cell carcinoma was essential to be explored further. In this study, we also observed a significant increase in the level of β-catenin protein in SeCC cases (*n* = 13/16, 81 %). These results indicated that the oncogenic potential of the W*nt*/β-catenin transduction pathway was related to the development of SeCC. However, specific β-catenin labeling was not observed in the nuclei of SeCC cells. It suggests that the mechanism of SeCC tumorigenesis was much different from that of human OSSN.

In addition to overexpression of β-catenin in our SeCC cases, we also demonstrated the expression of CRABPs and related RA receptors in SeCC cases. CRABPs binded all-trans-RA intracellularly and might be involved in the transfer process of RA into the cell nucleus. CRABP1 played a role in presenting RA to metabolizing (CYP26) enzymes, and CRABP2 played a role in transfer of RA to nuclear RARs by direct protein–protein interactions. In our data, the CRABP1 protein was not expressed strongly in the tumor cells of SeCC cases, whereas the CRABP2 protein was overexpressed in the tumor cells of SeCC cases. These results indicated that CRABP2 protein might play an important role in the pathoetiology of sebaceous cell carcinoma.

The FABPs belonged to a group of intracellular lipid chaperones that bind fatty acids, retinoids, and hydrophobic compounds, and mediate their biological functions [22]. In particular, FABP5 was the only one of the family to bind retinoic acid [12]. Therefore, CRABP1, CRABP2, and FABP5 were retinoid-binding proteins expressed in mammalian skin and appendages that were known to regulate RA signaling [11, 23]. In our data, the FABP5 protein was also aberrant expressed in some tumor cells of SeCC cases. Collins and Watt reported that CRABP1, CRABP2, and FABP5 proteins were dynamically expressed during skin development and in adult tissue [11]. Their findings demonstrated that there was dynamic regulation of RA signaling in different regions of the skin, and provided evidence for interactions between the RA, β-catenin, and Notch pathways [11]. Furthermore, they found that the CRABP1, CRABP2, and FABP5 proteins were overexpressed in both benign papillomas and malignant squamous cell carcinomas (SCCs) [11]. In particular, CRABP1 was expressed in the tumor stroma, and CRABP2 and FABP5 were expressed in the sebaceous gland cells, interfollicular epidermis, and hair follicles. Our results supported those molecules were also upregulated in the tumor cells of sebaceous cell carcinoma. In this study, we observed a significant increase in the level of expression of CRABP2 and FABP5 in SeCC cases compared with controls. CRABP2 and FABP5 were expressed in hair follicles of eyelid skin in both groups, whereas CRABP2 and FABP5 were aberrantly expressed in the SeCC tumor cells. In addition, we found CRABP2 and FABP5 were aberrantly expressed in severe SeCC patients and these results may be related to distant metastasis and poor prognosis (Case 16, Fig. 1d).

In 10 cases of sebaceous cell carcinoma, Chakravarti and co-workers found that three cases had decreased RAR-α expression (absent in three cases); six cases had increased RAR-β expression; expression was absent in four cases, and two cases had decreased RAR-γ expression compared with controls [24]. Moreover, RXR-α expression was decreased, RXR-β expression was low in seven tumors, and RXR-γ expression was absent in six tumors. They concluded that aberrant expression of retinoid receptors in sebaceous cell carcinoma of the eyelid might play a role in the pathogenesis and progression of this carcinoma [24]. In our study, RAR-β, RXR-β, and RXR-γ were predominantly expressed in SeCC cases, whereas RAR-α, RAR-γ, RXR-α were presented at lower levels in SeCC cases with no significant difference as compared with control cases. Further studies were needed to investigate the association of dysregulation of retinoic acid receptors and the prognosis of sebaceous cell carcinoma.

## Conclusions

Our study results indicate that the β-catenin, RA signaling binding proteins and related RA receptors are related to the development of sebaceous cell carcinoma. These results reveal that the retinoic acid binding proteins and related RA receptors may affect the growth of sebaceous cell carcinoma. Our findings indicate that retinoic acid signaling is related to the pathogenesis of sebaceous cell carcinoma of the eyelids. Further experiments are ongoing to study the treatment of sebaceous cell carcinoma with retinoic acid-related products.

## Consent

Written informed consent was obtained from the patients for publication and any accompanying images. A copy of the written consent is available for review by the Editor-in-Chief of this journal.

## Abbreviations

SeCC: Sebaceous cell carcinoma
RA: Retinoic acid
CRABP1: Cellular retinoic acid binding protein 1
CRABP2: Cellular retinoic acid binding protein 2
FABP5: Fatty acid-binding protein 5
RAR-α, -ß, -γ: Retinoic acid receptors-α, −β, −γ
RXR-α, -β, -γ: Retinoid X receptors-α, −β, −γ
